# Supplementary material for: Care of peripheral intravenous catheters in three hospitals in Spain: Mapping clinical outcomes and implementation of clinical practice guidelines
Source: PLoS One. 2020 Oct 2;15(10):e0240086. doi: 10.1371/journal.pone.0240086 (PMC7531784; doi:10.1371/journal.pone.0240086)
Supplement: S1 Table — (PDF) [file pone.0240086.s001.pdf]

Supporting Table 1. Ratings on a 20-Item case report form by clinical experts.

Item	Expert 1	Expert 2	Expert 3	Expert 4	Expert 5	Expert 6	Number in agreement	Item CVI
Patient characteristics								
Age	x	x	x	x	x	x	6	1.0
Gender	x	x	x		x		4	0.57
Cognitive impairment	x	x	x	x	x	x	6	1.0
Patient knowledges related to PIVC	x	x	x	x	x	x	6	1.0
catheter adequacy and insertion								
Vascular access device type	x	x	x	x	x	x	6	1.0
Insertion site	x	x	x	x	x	x	6	1.0
Intravenous cannula size	x	x	x	x	x	x	6	1.0
Indwelling time	x	x	x	x	x	x	6	1.0
Setting of Insertion	x	x	x	x	x	x	6	1.0
Catheter and catheter site care								
Inspection of insertion site	x	x	x	x	x	x	6	1.0
Securement	x	x	x	x	x	x	6	1.0
Dressing type	x	x	x	x	x	x	6	1.0
Dressing status	x	x	x	x	x	x	6	1.0
add-on devices or connectors	x	x	x	x	x	x	6	1.0
Infusion type	x	x	x	x	x	x	6	1.0
Catheter removal and replacement strategies								
Visualization of adverse event	x	x	x	x	x	x	6	1.0
PIVC failure	x	x	x	x	x	x	6	1.0
Unnecessary PIVC	x	x	x	x	x	x	6	1.0
Record and documentation PIVC care								
PIVC insertion records	x	x	x	x	x	x	6	1.0
Dressing date recorded	x	x	x		x	x	5	0.83
**Proportion relevant**	1.0	1.0	1.0	0.9	1.0	0.95	**Mean I-CVI**	0.97
							**S-CVI/UA**	0.90
							**Mean expert proportion**	0.98
Table 2. Ratings on a 25-Item case report form by clinical experts.								

I-CVI, item-level content validity index.

S-CVI/UA, scale-level content validity index, universal agreement calculation method.
